# Profile of patients with cleft palate fitted with hearing AIDS

**DOI:** 10.1016/S1808-8694(15)30555-3

**Published:** 2015-10-19

**Authors:** Ticiana Cristina de Freitas Zambonato, Mariza Ribeiro Feniman, Wanderléia Quinhoneiro Blasca, José Roberto Pereira Lauris, Luciana Paula Maximino

**Affiliations:** 1 MSc in Rehabilitation Sciences, Hospital of Rehabilitation and Craniofacial Anomalies at the University of São Paulo, HRAC-USP. Speech and Hearing Therapist; 2 Associate Professor, Department of Speech and Hearing Therapy, Bauru Dental School, University of São Paulo, FOB-USP; 3 PhD in Communication Disorder Sciences, Hospital of Rehabilitation and Craniofacial Anomalies at the University of São Paulo, HRAC-USP. Professora, Department of Speech and Hearing Therapy, Bauru Dental School, University of São Paulo, FOB-USP; 4 Associate Professor, Department of Dental Pediatrics, Orthodontics, and Collective Health, Bauru Dental School, University of São Paulo, FOB/USP; 5 PhD in Biologic Sciences - Human Genetics, UNESP at Botucatu; Professor, Speech and Hearing Therapy Department, Bauru Dental School, University of São Paulo FOB/USP. Hospital de Reabilitação de Anomalias Craniofaciais da Universidade de São Paulo, HRAC-USP

**Keywords:** cleft palate, hearing loss, hearing loss, mixed conductive-sensorineural, conductive

## Abstract

Cleft palates cause alterations in palate and lip structures, and it may also cause hearing loss because of recurrent otitis media. The appropriate treatment is controversial. It may include the prescription of antibiotics and insertion of a ventilation tube, or even otorhinolaryngological and audiological assistance, and hearing rehabilitation, with the use of an individual sound amplifier aid (ISAA).

**Aim:**

To characterize the profile of individuals with cleft palate and hearing loss, users of ISAA are assisted by the center of otorhinolaryngology and speech therapy of a hospital specialized in craniofacial anomalies and hearing impairment. Retrospective Study.

**Materials and Methods:**

Retrospective analysis of 131 charts of patients with corrected cleft palate and hearing loss, fitted with ISAA by the center abovementioned.

**Results:**

The sample (n=131) was characterized by a prevalence of females (53%), unilateral incisive transforaminal cleft (27%), presence of associated anomalies (51%), history of alterations of the middle ear (56%) and surgery intervention (56%).

**Conclusion:**

The general profile of the individuals with cleft palate and hearing loss, fitted with ISAA, was characterized by the predominance of cleft lip and palate, positive history of middle ear alterations, surgery intervention and bilateral sensorineural hearing loss.

## INTRODUCTION

Cleft lip and palate is a malformation that involves the lip and palate structures leading to possible oral communication disorders, principally due to audiological changes and alterations in the speech-related joints, apart from introducing feeding, psychosocial, educational, dental, and cosmetic disorders.^1^

Cleft lip and palate patients present middle ear ventilation problems resulting from changes in the movement of the Eustachian tube caused by inadequate insertion of the palate tensor and elevator muscles, thus producing a functional obstruction on the Eustachian tube and negative pressure in the middle ear, leading to otitis media.^2^,^3^

The significant degree of association between cleft lip and palate and changes in the middle ear has been profusely described in the literature^2^,^4^, ^5^, ^6^, ^7^, ^8^, ^9^, ^10^, ^11^, ^12^ with wide agreement among authors. The most adequate therapy mode for this disorder remains yet controversial.

Various studies have supported the importance of using antibiotics and inserting the Eustachian tube during palate repair surgery to prevent the occurrence of middle ear disorders and their consequences.^10^,^13^, ^14^, ^15^, ^16^, ^17^ Other studies^2^,^7^,^8^,9,^18^,^19^ reported high rates of long term side effects such as perforation and retraction of the tympanic membrane, chronic otitis media, and hearing loss in the ears treated with tube ventilation insertion. Ideal treatment was then defined as otorhinolaryngological and audiological follow-up, accompanied by aural rehabilitation and use of hearing aids. The indication to insert a ventilation tube is only maintained after thoroughly discussing the matter with the patient and/or his/her guardian and explaining the possible consequences of the procedure when objective evidences of persistent effusion are present for over 3 months.

The use of hearing aids as a mode of aural rehabilitation in cleft lip and palate patients with hearing loss is a viable and relevant alternative for such individuals. It is worth mentioning that this indication requires a complex process through which the right device is chosen and validated for each patient^20^,^21^.

In such context, given the relevance of the association between cleft lip and palate and hearing loss, this study aims to characterize the profile of patients with cleft lip and palate and hearing loss using hearing aids.

## MATERIALS AND METHOD

This study was approved by the Ethics Committee under permit 314/2005UEPCEP. A retrospective study was carried out on the charts of 131 patients operated for cleft lip and palate^27^,^28^ and offered hearing aids between 2001 and 2006.

This research was carried out between August of 2005 and August of 2007.

Patient charts were checked for data on patient identification (gender, date of birth, birthplace, degree of education, type of cleft lip and palate), otologic history (history of otitis, surgery), hearing (most recent audiological assessment) and hearing aid (type and model).

The classification developed by Spina et al. and modified by Silva Filho et al. was used to describe the cleft lip and palate types. This method uses the incisive foramen - the point where lip and palate are joined together - as an anatomic landmark; for clarification purposes, however, as results are presented in our study, the chosen nomenclature was cleft lip for pre-incisive foramen manifestations, cleft palate for post-incisive foramen occurrences, and cleft lip and palate for trans-incisive foramen alterations.

Results were submitted to a descriptive analysis after data collection and summarized in the form of tables to facilitate analysis and interpretation. The chi-square test was performed with a significance level of 5% (p<0.05) to check for the association between the analyzed variables.

All patients in our sample are being treated and followed specifically for their craniofacial malformations and systematically followed by the Otorhinolaryngology and Speech and Hearing Therapy service at a hospital specialized in craniofacial anomalies and hearing loss.

## RESULTS

Patients were aged between 2 and 72 years (mean=23; median=20; standard deviation=14.7).

Table 1 shows the distribution of the sample according o gender and cleft type.

**Table 1:** Patient distribution according to gender and cleft type

Cleft type	Gender		TOTAL(%)
	Male (%)	Female (%)	
Lip	2(22)	7(78)	9(100)
Palate	20(38)	32(62)	52(100)
Lip+Palate	40(57)	30(43)	70(100)
TOTAL	62(47)	69(53)	131(100)

X^2^= 6,62;p = 0,033*

The presence of anomalies associated with cleft lip and palate was analyzed in all charts, and 67 (51%) patients were found to have other anomalies associated with cleft lip and palate.

The otologic history of the studied population is shown in Table 2, Table 3, on history of otitis and surgery respectively. Among the surgical procedure performed are myringotomy to insert the ventilation tube, tympanoplasty, tympanomastoidectomy, and radical mastoidectomy.

**Table 2:** Patient distribution according to cleft type and history of otitis

Cleft Type	Otitis History		TOTAL(%)
	Yes (%)	No (%)	
Lip	3 (33)	6 (67)	9 (100)
Palate	27 (52)	25 (48)	52 (100)
Lip + Palate	32 (46)	38 (54)	70 (100)
TOTAL	62(47)	69(53)	131(100)

X2 = 1,22; p = 0,543 ns

**Table 3:** Patient distribution according to cleft type and presence of otitis and surgery

Cleft type	Otitis and surgery				TOTAL(%)
	A(%)	B(%)	C(%)	D(%)	
Lip	1 (11)	2 (22)	1 (11)	5 (56)	9 (100)
Palate	16 (31)	11 (21)	4 (8)	21 (40)	52 (100)
Lip + Palate	24 (34)	19 (27)	8 (11)	19 (27)	70 (100)
TOTAL	41(31)	32(25)	13(10)	45(34)	131(100)

X2 = 5,05; p = 0,537 ns Legend: A - otitis with surgery; B - otitis without surgery; C - surgery without otitis; D - neither otitis nor surgery

Table 4 comprises the data on hearing as found in the most recent audiological assessments performed on the patients; patients are grouped as a function of the type of lip and palate malformation.

**Table 4:** Patient distribution according to cleft type, normal hearing, and hearing loss type per ear

Cleft Type	Right Ear	Left Ear				TOTAL (%)
		Normal	CD	MX	SN	
Lip	Normal					
	CD					
	MX			2	1	3 (33)
	SN				6	6 (67)
	Total(%)			2 (22)	7 (78)	9 (100)
	Normal		1	1	7	9 (13)
	CD	3	7	2	1	13 (19)
Palate	MX			16	3	19 (27)
	SN	2	1	5	21	29 (41)
	Total(%)	5 (7)	9 (13)	24 (34)	32 (46)	70 (100)
	Normal					
	CD	2	15	2		19 (37)
Lip + Palate	MX	1	2	9		12 (23)
	SN	1	1	2	17	21 (40)
	Total(%)	4(3)	18(14)	13(10)	17(13)	52 (100)

Legend: CD - conductive hearing loss; MS - mixed hearing loss; SN - sensorineural hearing loss

Use of hearing AID per type is described in Table 5.

**Table 5:** Patient distribution according to hearing aid type per ear

HA Right Ear	HA Left Ear								TOTAL (%)
	NF	L-R	L-ITC	NL-R	NL-ITC	NL-CIC	R-B	C-B	
NA		10	1	5		3	2	2	23 (18)
L-R	4	43							47 (36)
NL-R	10	4		22					36 (27)
NL-ITC					1				1 (1)
NL-CIC	2					14			16 (12)
R-BT	2								2 (2)
C-BT	5							1	6 (5)
TOTAL (%)	23 (18)	57 (44)	1 (1)	27 (21)	1 (1)	17 (13)	2 (2)	3 (2)	131 (100)

Legend: NF - not fitted; L-R - linear retro; L-ITC - linear intracanal; NL-R - non-linear retro; NL-ITC - non-linear intracanal; NL-CIC - non-linear micro canal;R-B - retro bone transmission; C-B - conventional bone transmission

## DISCUSSION

Our study looked into the charts of 131 patients with cleft lip and palate fitted with hearing aids, with ages ranging between 2 and 72 years, most of which females. According to the literature, isolated cleft palates are more common in women, as also found in our study (Table 1), in which 62% of the female patients had cleft palates^29^, a statistically significant difference among genders.

As vastly described in the literature,^4^,^5^,^6^,^9^,^10^,^16^,^30^ this study (Table 2) also saw a large portion of cleft palate patients with history of middle ear disorders.

Most patients with cleft lip (67%) do not have a history of otitis; in contrast, 52% of the patients with cleft palate have a history of otitis (Table 2), although no statistically significant differences were found (p=0.543).

Another study reported worse air tone thresholds in patients with cleft palates when compared to cleft lip patients, indicating a greater probability of hearing loss in cleft palate patients due to middle ear disorders.^31^ Similar findings can be seen in Table 3, in which patients with cleft lip and palate had a larger occurrence of otitis and surgery than others, despite the lack of statistical significance (p=0.537).

When looking at hearing (Table 4), all types of hearing loss (conductive, mixed and sensorineural) were present in the sampled population, as well as absence of hearing involvement. It may be seen, after analyzing hearing and type of malformation, that cleft lip patients have more sensorineural hearing loss. This finding is probably related to other etiologic factors and not specifically to the cleft deformity, as only the lip is involved in this malformation. In contrast, in cleft palate alone and in cleft lip and palate cases other types of hearing loss were found, possibly justifying the presence of some important conductive component. Our findings depict structural and functional changes introduced by the occurrence of cleft palate, such as Eustachian tube disorders and middle ear infections, apart from increased rates of respiratory tract infections, hypertrophic tonsil and adenoids, and immune disorders connected to lack of breastfeeding during infancy.^2^,^3^,^4^,^5^,^6^,^9^,^10^,^15^,^32^,^33^

The prevalence of otitis media was reduced as a result of palate repair surgery, but it is possible that middle ear disorders linger on for a long time, until the adulthood.^9^,^10^,^30^,^34^ Some authors associate the persistence of otitis media after palate repair and functional disorder to patient predisposition to secondary diseases introduced by primary middle ear infections.^35^

Fifty six percent of the patients enrolled in our study underwent surgery to treat recurring otitis media, showing a greater tendency towards surgical treatment as also seen in the literature as a preventive approach to middle ear disorders.^10^,^14^, ^15^, ^16^, ^17^

However, many discuss the possibility of offering patients a less aggressive therapy that does not introduce late complications, provided in the form of otorhinolaryngological and audiological follow-up combined with indication of hearing aids.^1^,^2^,^5^,^7^,^8^,^11^,^18^,^35^, ^36^, ^37^, ^38^, ^39^, ^40^, ^41^, ^42^

Some authors state that the use of hearing aids is an effective tool in treating persistent and recurring otitis media after the ventilation tube has been removed. Additionally, they found that when the justification to treat otitis is hearing loss and the ensuing disabilities, hearing aids can be used as an effective non-invasive therapeutic measure.^40^

## CONCLUSION

Our sample of patients with cleft lip and palate and hearing loss fitted with hearing aids may be characterized mainly as patients with cleft lip and palate with a history of middle ear disorders treated surgically in cases of recurring otitis media.
